# Avian influenza virus detection, temporality and co-infection in poultry in Cambodian border provinces, 2017–2018

**DOI:** 10.1080/22221751.2019.1604085

**Published:** 2019-04-19

**Authors:** Erik A. Karlsson, Srey Viseth Horm, Songha Tok, Sothyra Tum, Wantanee Kalpravidh, Filip Claes, Kristina Osbjer, Philippe Dussart

**Affiliations:** a Virology Unit, Institute Pasteur du Cambodge, Phnom Penh, Cambodia; b National Animal Health and Production Research Institute, Cambodian Ministry of Agriculture, Forestry and Fisheries, Phnom Penh, Cambodia; c Regional Office for Asia and the Pacific, Food and Agriculture Organization of the United Nations, Bangkok, Thailand; d Emergency Centre for Transboundary Animal Diseases, Food and Agriculture Organization of the United Nations, Phnom Penh, Cambodia

Highly pathogenic avian influenza virus (AIV) has been endemic in Cambodia since 2004, and is a major agricultural and public health concern. Cambodia is a tropical, resource poor, lower-middle income country in Southeast Asia with a large socio-economic dependence on agriculture. In 2015, 87% of Cambodian households with agricultural holdings raised poultry [1], mainly on small, backyard farms with minimal biosafety and/or biosecurity. In conjunction with the National Animal Health and Production Institute (NaHPRI), Institut Pasteur du Cambodge (IPC) has maintained active longitudinal surveillance at key live bird markets (LBMs) in the heavily populated, southern part of the country [2–4]. Cambodian LBMs have high levels of AIV circulation, with 30–50% of ducks and 20–40% of chickens testing positive [3,4]. Concerningly, a multitude of high and low pathogenic AIVs circulate concurrently. Previous studies suggest peak AIV circulation corresponds to the dry season (November to May) especially around Lunar New Year (LNY) celebrations when poultry consumption is highest [3–6]. Although we have comprehensive, longitudinal data on AIV circulation in key Cambodian LBMs, there is a distinct lack of knowledge about AIVs in rural areas. Border regions with Vietnam and Thailand are particularly crucial for improved surveillance, as cross-border routes are key factors in novel AIV introduction [5]. Therefore, we performed longitudinal surveillance in three Cambodian border provinces between 2017 and 2018, focusing on national festivals where poultry trade and consumption is increased, to understand circulation, prevalence and temporality of AIV in domestic poultry in these key regions.

Between August 2017 and May 2018, 16 total sampling sessions were performed in LBMs, slaughterhouses and poultry storage facilities in Takeo, Kandal and Banteay Meanchey (BM) provinces, concurrently. Sampling on the week before, the week of, and the week after the four major Cambodian festival periods: Pchum Ben (PB; September 2017), Bon Om Touk (BOT; Water Festival; November 2017), LNY (February 2018) and Khmer New Year (KNY; April 2018) with single samplings in-between (Supplemental Figure 1). One tracheal and one cloacal swab were collected and pooled into a single tube from at least 40 individual birds/province/sampling for a total of 2129 poultry samples and tested for AIV by RT-qPCR as previously described [3,4]. While a few samples from domestic turkeys (*Meleagris gallopavo*) and geese (*Anser anser domesticus*) were taken, the majority were from domestic chickens (*Gallus gallus domesticus*) and ducks (*Anatidae spp*), so analysis focused on these two poultry types.

Overall, 23.3% of the poultry samples screened were positive for AIV by RT-qPCR with 20.0% and 32.6% positivity in chickens and ducks, respectively. Percentages were similar for individual provinces (Supplemental Table 1). Longitudinally, total AIV detection fluctuated between 4.0% and 48.3%, with highest levels the week before or the week of festivals with increased poultry consumption. Highest detection was associated with LNY followed closely by KNY (Figure 1(A)). Similar patterns were observed in individual provinces (Figure 1(B–D)). By subtype, 25.2%, 7.4%, 52.3%, 18.1% of AIV positive poultry samples were subtyped as A/H5, A/H7, A/H9, and “unknown,” respectively (Supplemental Table 1). The majority (42.3%) of AIV samples from ducks were positive for subtype A/H5 versus 15.2% in chickens. Similarly, 19.8% and 30.8% of AIV positive ducks were positive for A/H7 and unknown subtype versus 0.32% and 10.8% in chickens, respectively. Conversely, 76.2% AIV positive chickens were of A/H9 subtype versus 11.0% of ducks (Supplemental Figure 2(A)). A/H5 detection peaked during LNY in accordance with previous studies [3,4]. Peaks were also observed during PB and BOT; however, detection was minimal during KNY. Interestingly, A/H9 detection was high during festival periods but A/H7 peaked between LNY and KNY (Figure 1(E)). A/H9 dominated in chickens whereas A/H5 dominated in ducks (Supplemental Figure 2(E,I)). By province, A/H5 detection reflected total samples. Outside of festival periods, Kandal and BM provinces were dominated by A/H9 and unknown subtypes whereas Takeo had subtype “waves,” including A/H7 (Figure 1(F–H)). Chickens from Kandal and BM were dominated by waves of A/H9 and A/H5, whereas Takeo also had waves of unknown subtypes (Supplemental Figure 3(B–D)). In all provinces, 100% of AIV positive duck samples were subtyped as A/H9 during PB and for A/H5 the week before LNY. Otherwise, peaks were inconsistent between provinces (Supplemental Figure 3(E–H)).

**Figure 1.**
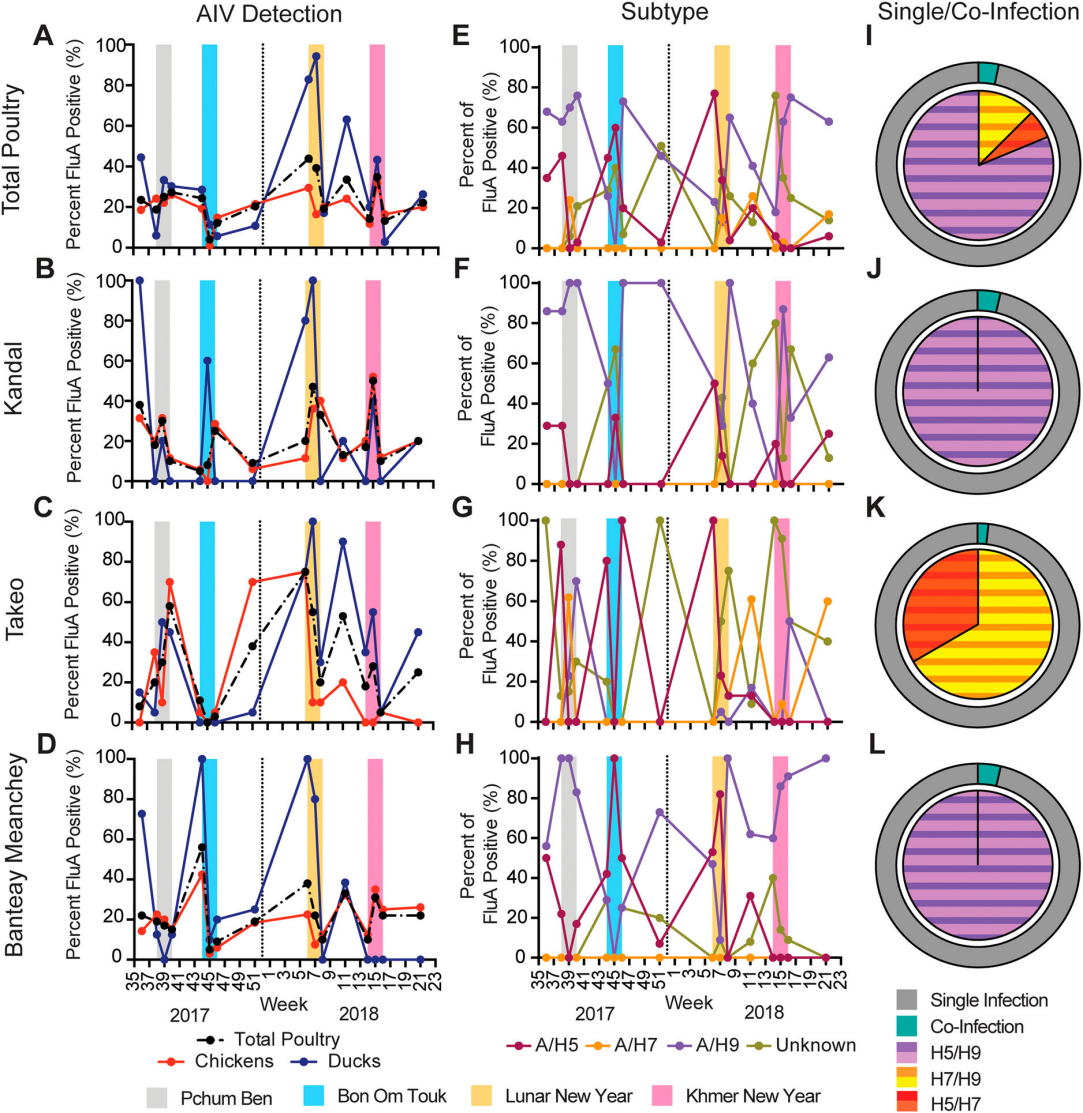
Detection of influenza A viruses in total poultry in border provinces of Cambodia. Notes: (A–D) Per cent influenza A virus positive samples in total poultry (dashed line), chicken, and duck samples in all provinces combined (A), as well as Kandal (B), Takeo (C), and Banteay Meanchey (D) provinces individually. (E–H) Per cent detection of subtype of influenza A virus positive samples by sampling visit for A/H5, A/H7, A/H9, and unknown subtype from total poultry for all provinces combined (E) as well as in samples from Kandal (F), Takeo (G) and Banteay Meanchey (H) provinces individually. Major festivals are indicated as Pchum Ben, Bon Om Touk (Water Festival), Lunar New Year, and Khmer New Year. Vertical dashed line indicates split between 2017 and 2018. (I–L) Per cent of influenza A positive samples positive for co-infections (outer ring) from total poultry for all provinces combined (I) as well as in samples from Kandal (J), Takeo (K) and Banteay Meanchey (L) provinces individually. Co-infections were classified into combinations of A/H5–H9 subtypes (inner circle), A/H5–H7 subtypes (inner circle), and A/H7–H9 subtypes (inner circle).

Co-infections comprised 3.2% of all AIV positive samples (Figure 1(I)). The majority (86.7%) of co-infections were classified as A/H5 + A/H9; however, co-infections between A/H5 + A/H7 and A/H7 + A/H9 were also detected in 6.7% and 13.3% of total co-infections, respectively. Prevalence of co-infection was similar between chickens (3.2%) and ducks (3.3%); however, co-infections in chickens were exclusively the A/H5 + A/H9 combination whereas ducks had a higher diversity with 50%, 16.7% and 33.3% of co-infections identified as A/H5 + A/H9, A/H5 + A/H7, and A/H7 + A/H9, respectively (Supplemental Figure 4(A,E)). Co-infections were detected in both chickens (2.8%) and ducks (13.6%) in Kandal, but only in chickens (4.8%) in BM and only in ducks (2.7%) in Takeo (Supplemental Figure 4(B–D,F–H)). The greatest prevalence of co-infections were detected at week 11 of 2018 at 5.7% of total AIV positive samples (Supplemental Figure 4(I)).

As observed previously [4], A/H5 isolates that could be subtyped were also positive for neuraminidase (NA) subtype N1 by RT-qPCR. No novel H5Nx viruses were detected. A/H9 isolates were subtyped with N2 and A/H7 samples were identified with the N7 and N4 NA subtype by conventional PCR. All A/H7 samples are of the Eurasian lineage, and, to date, AIV similar to the A/Anhui/1/2013-lineage have not been detected in Cambodia. While some samples could not be typed due to low viral load, no positive samples were subtyped for N3, N6, N8 or N9 by RT-qPCR or conventional methods.

Overall, AIV continues to circulate within Cambodia at high levels as previously described, correlating to festival periods when poultry production and consumption is increased [2–4]. However, a human infection has not been detected since 2014. Border regions display variable AIV prevalence and diversity, possibly due to poultry movement across borders. While A/H7 was detected previously in Cambodia [4,7], subtype A/H7N4 presents concern due to the human case in nearby China at a similar time period [8]. In addition, detection of co-infections in 3.2% of AIV positive poultry, especially with A/H9, raises concerns about reassortment and emergence of novel viruses with epizootic or pandemic potential [9]. Isolation, Whole Genome Sequencing, and unknown subtype determination is on-going to further characterize these viruses on a molecular and phylogenetic level. Continued, active, vigilant surveillance is vital and interventions to decrease the prevalence of AIVs in LBMs should be considered, especially during festival periods.

## Supplementary Material

Supplemental Material
