# Supplementary material for: Avian influenza virus detection, temporality and co-infection in poultry in Cambodian border provinces, 2017–2018
Source: Emerg Microbes Infect. 2019 Apr 19;8(1):637–9. doi: 10.1080/22221751.2019.1604085 (PMC6493305; doi:10.1080/22221751.2019.1604085)
Supplement: Supplemental Material [file TEMI_A_1604085_SM1115.zip › temi-2019-0074-20190404174520/graphic/doc/LBM1718_EMI_SuppTable_FINAL.docx]

| Province | Poultry Type | Total n | Flu A  (M-gene) | | A/H5 | | A/H7 | | A/H9 | | Unknown | | A/H5  +A/H9 | | A/H5  +A/H7 | | A/H7  +A/H9 | |
| --- | --- | --- | --- | --- | --- | --- | --- | --- | --- | --- | --- | --- | --- | --- | --- | --- | --- | --- |
|  |  |  | n | % Total | n | % FluA | n | % FluA | n | % FluA | n | % FluA | n | %  FluA | n | %  FluA | n | %  FluA |
| Kandal | Chickens | 541 | 108 | 20.0% | 10 | 9.3% | 1 | 0.9% | 91 | 84.3% | 9 | 8.3% | 3 | 2.8% | 0 | 0.0% | 0 | 0.0% |
|  | Ducks | 80 | 22 | 27.5% | 11 | 50.0% | 1 | 4.5% | 5 | 22.7% | 8 | 36.4% | 3 | 13.6% | 0 | 0.0% | 0 | 0.0% |
|  | Total | 621 | 130 | 20.9% | 21 | 16.2% | 2 | 1.5% | 96 | 73.8% | 17 | 13.1% | 6 | 4.6% | 0 | 0.0% | 0 | 0.0% |
| Takeo | Chickens | 320 | 63 | 19.7% | 27 | 42.9% | 0 | 0.0% | 18 | 28.6% | 18 | 28.6% | 0 | 0.0% | 0 | 0.0% | 0 | 0.0% |
|  | Ducks | 320 | 111 | 34.7% | 23 | 20.7% | 35 | 31.5% | 13 | 11.7% | 43 | 38.7% | 0 | 0.0% | 1 | 0.9% | 2 | 1.8% |
|  | Total | 640 | 174 | 27.2% | 50 | 28.7% | 35 | 20.1% | 31 | 17.8% | 61 | 35.1% | 0 | 0.0% | 1 | 0.6% | 2 | 1.1% |
| Banteay Meanchey | Chickens | 710 | 144 | 20.3% | 11 | 7.6% | 0 | 0.0% | 131 | 91.0% | 7 | 4.9% | 7 | 4.9% | 0 | 0.0% | 0 | 0.0% |
|  | Ducks | 158 | 49 | 31.0% | 43 | 87.8% | 0 | 0.0% | 2 | 4.1% | 5 | 10.2% | 0 | 0.0% | 0 | 0.0% | 0 | 0.0% |
|  | Total | 868 | 193 | 22.2% | 54 | 28.0% | 0 | 0.0% | 133 | 68.9% | 12 | 6.2% | 7 | 3.6% | 0 | 0.0% | 0 | 0.0% |
| Combined | Chickens | 1571 | 315 | 20.1% | 48 | 15.2% | 1 | 0.3% | 240 | 76.2% | 34 | 10.8% | 10 | 3.2% | 0 | 0.0% | 0 | 0.0% |
|  | Ducks | 558 | 182 | 32.6% | 77 | 42.3% | 36 | 19.8% | 20 | 11.0% | 56 | 30.8% | 3 | 1.6% | 1 | 0.5% | 2 | 1.1% |
|  | Total | 2129 | 497 | 23.3% | 125 | 25.2% | 37 | 7.4% | 260 | 52.3% | 90 | 18.1% | 13 | 2.6% | 1 | 0.2% | 2 | 0.4% |

**Supplemental Table 1:** Number (n) and Percent (%) Detection of Influenza A Virus is Chicken and Duck Samples from Kandal, Takeo, and Banteay Meanchey Provinces, Cambodia between August 2017 and May 2018.
