# Supplementary material for: Avian influenza virus detection, temporality and co-infection in poultry in Cambodian border provinces, 2017–2018
Source: Emerg Microbes Infect. 2019 Apr 19;8(1):637–9. doi: 10.1080/22221751.2019.1604085 (PMC6493305; doi:10.1080/22221751.2019.1604085)
Supplement: Supplemental Material [file TEMI_A_1604085_SM1115.zip › temi-2019-0074-20190404174520/graphic/LBM1718_EMI_SuppFigures_FINAL.docx]

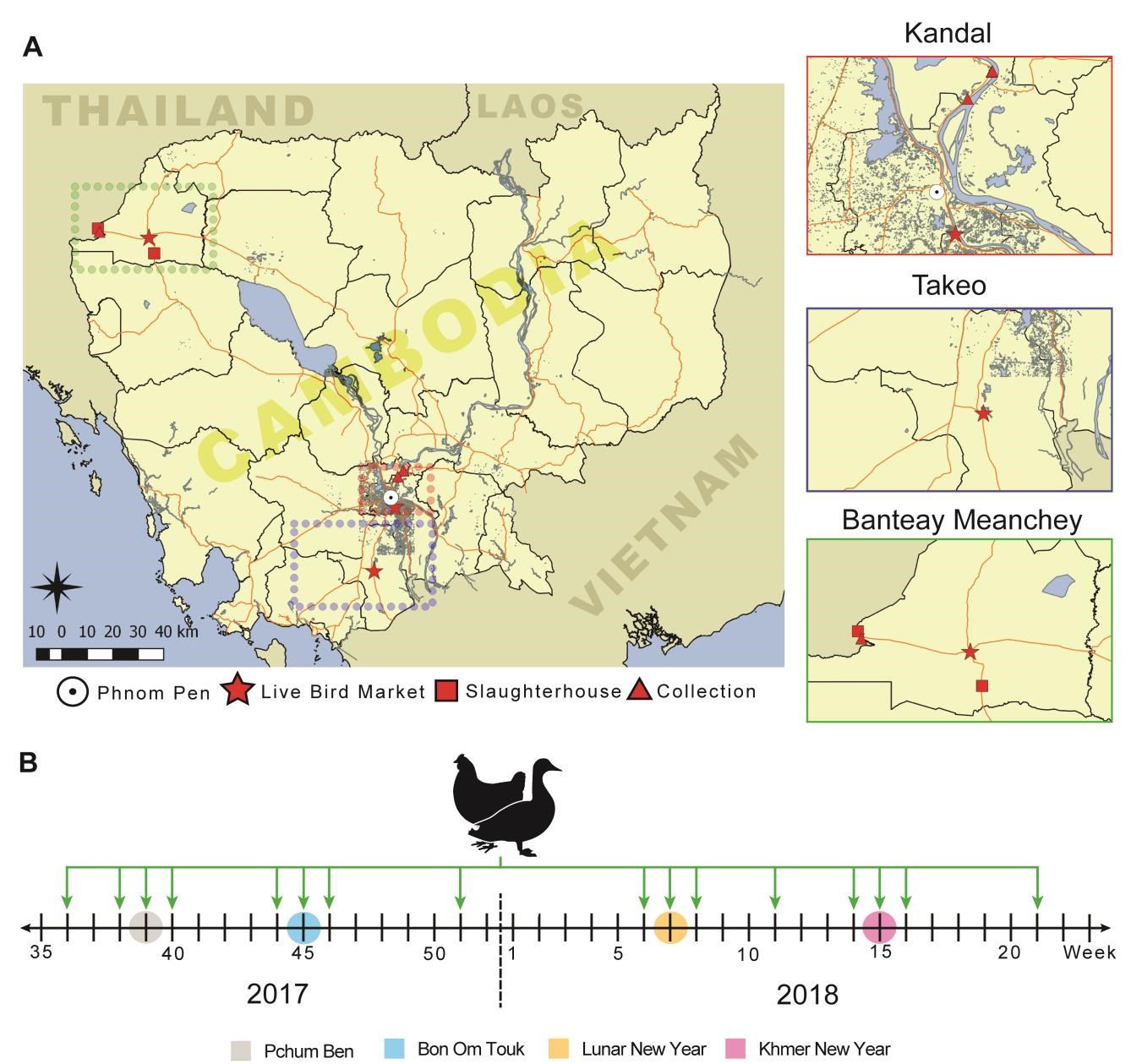
**Supplemental Figure 1: Sampling Sites and Timeline**. (A) Map of the sampling sites chosen in Kandal (red box), Takeo (blue box) and Banteay Meanchey (green box) provinces. Poultry sampling sites are designated in red with storage facilities designated by triangles, slaughterhouses by squares, and live poultry markets by stars. (B) Timeline of sampling between August 2017 (week 36) and May 2018 (week 21). Sampling times are indicated with green arrows. Major festivals are indicated as Pchum Ben (gray), Bon Om Touk (Water Festival; blue), Lunar New Year (gold), and Khmer New Year (pink).


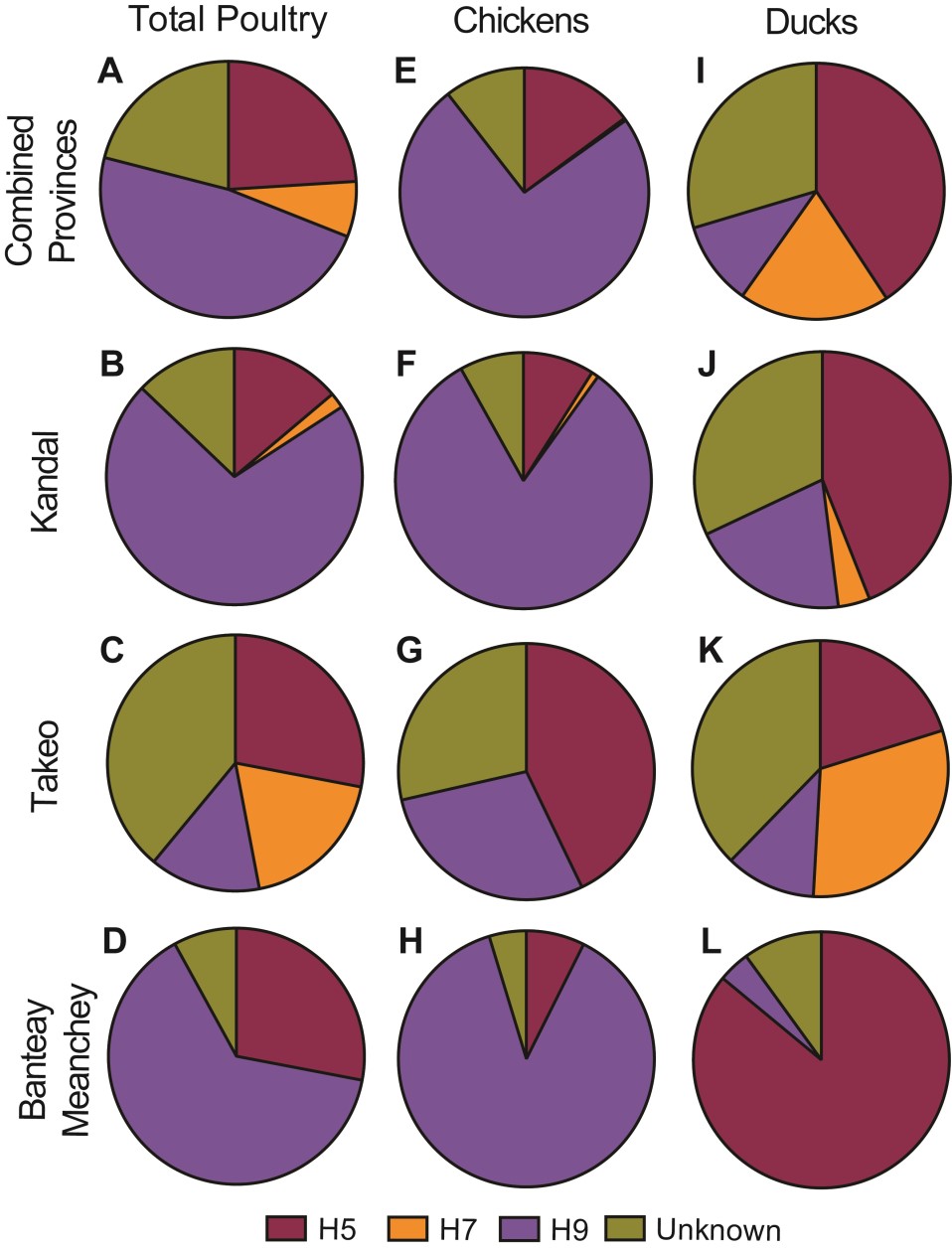
**Supplemental Figure 2**: Percent of influenza A positive samples positive for A/H5 (maroon), A/H7 (orange), A/H9 (purple), and unknown subtype (green) total poultry (A-D), chickens (E-H), and ducks (I-L). Data are presented for all provinces combined (A,E,I) as well as in samples from Kandal (B,F,J), Takeo (C,G,K) and Banteay Meanchey (D,H,L) provinces individually.


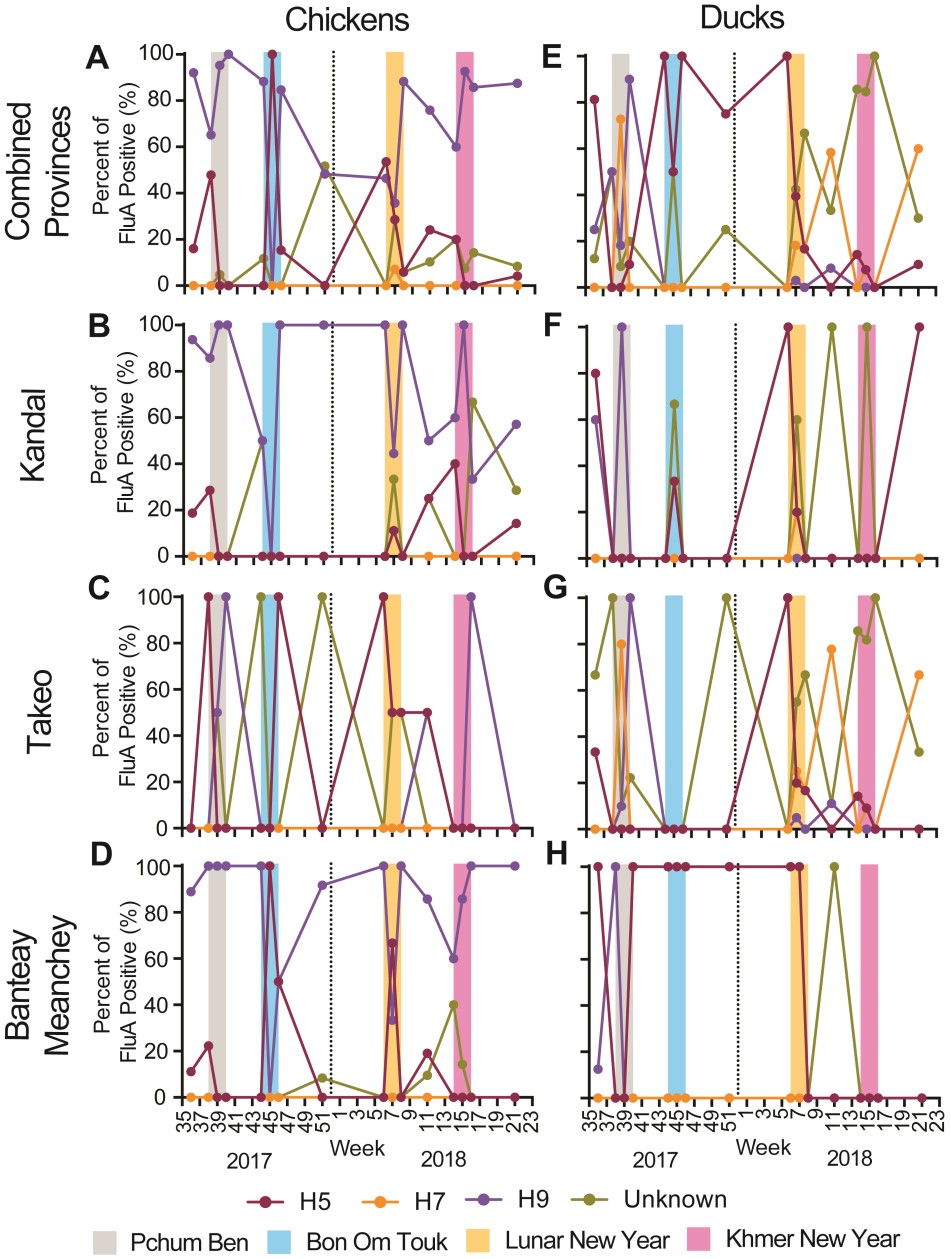
**Supplemental Figure 3**: Percent detection of subtype of influenza A virus positive samples by sampling visit for A/H5 (maroon), A/H7 (orange), A/H9 (purple), and unknown subtype (green) from chickens (A-D) and ducks (E-H) for all provinces combined (A,E) as well as in samples from Kandal (B,F), Takeo (C,G) and Banteay Meanchey (D,H) provinces individually. Major festivals are indicated as Pchum Ben (gray), Bon Om Touk (Water Festival; blue), Lunar New Year (gold), and Khmer New Year (pink). Vertical dashed line indicates split between 2017 and 2018.


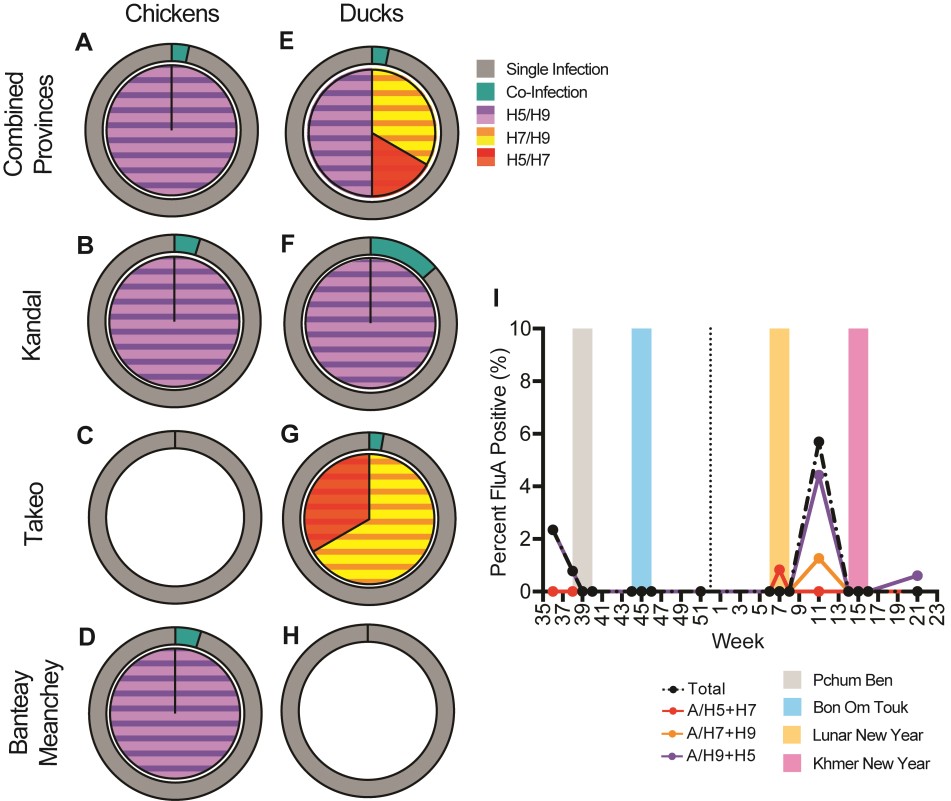
**Supplemental Figure 4**: Percent of influenza A positive samples positive for co-infections (teal; outer ring) from chickens (A-D) and ducks (E-H) for all provinces combined (A,E) as well as in samples from Kandal (B,F), Takeo (C,G) and Banteay Meanchey (D,H) provinces individually. Coinfections were classified into combinations of A/H5-H9 subtypes (purple; inner circle), A/H5-H7 subtypes (red; inner circle), and A/H7-H9 subtypes (yellow; inner circle). (I) Percent of influenza A virus positive samples positive for co-infections (black dashed line), and combination of A/H5 and A/H9 (purple line), combination of A/H5 and A/H7 (red line), and combination of A/H7 and A/H9 subtypes (orange line). Samples are from all provinces combined. Major festivals are indicated as Pchum Ben (gray), Bon Om Touk (Water Festival; blue), Lunar New Year (gold), and Khmer New Year (pink). Vertical dashed line indicates split between 2017 and 2018.
